# Survey indicated that core outcome set development is increasingly including patients, being conducted internationally and using Delphi surveys

**DOI:** 10.1186/s13063-018-2493-y

**Published:** 2018-02-17

**Authors:** Alice M. Biggane, Lucy Brading, Philippe Ravaud, Bridget Young, Paula R. Williamson

**Affiliations:** 1 Department of Biostatistics, University of Liverpool, Liverpool, UK; 2 INSERM, U1153 Epidemiology and Biostatistics Sorbonne Paris Cité Research Center (CRESS), Methods of therapeutic evaluation of chronic diseases Team (METHODS), 75014 Paris, France; 3 Paris Descartes University, Sorbonne Paris Cité, Paris, France; 4 Institute of Psychology Health and Society/North West Hub for Trials Methodology Research, University of Liverpool, Liverpool, UK; 5 Centre de Recherche Epidémiologie et Statistique, INSERM U1153, Paris, France; 6 Cochrane France, Paris, France; 7 Centre d’Épidémiologie Clinique, Hôpital Hôtel-Dieu, Assistance Publique-Hôpitaux de Paris, 75004 Paris, France; 8 Department of Epidemiology, Mailman School of Public Health, Columbia University, New York, NY USA; 9 Department of Psychological Sciences and MRC North West Hub for Trials Methodology Research, University of Liverpool, Liverpool, UK; 10 MRC North West Hub for Trials Methodology Research, Department of Biostatistics, University of Liverpool, Liverpool, UK

**Keywords:** Delphi, Survey, Patient engagement, Patient participation, Core outcome sets

## Abstract

**Background:**

There are numerous challenges in including patients in a core outcome set (COS) study, these can vary depending on the patient group. This study describes current efforts to include patients in the development of COS, with the aim of identifying areas for further improvement and study.

**Methods:**

Using the COMET database, corresponding authors of COS projects registered or published from 1 January 2013 to 2 February 2017 were invited via a personalised email to participate in a short online survey. The survey and emails were constructed to maximise the response rate by following the academic literature on enhancing survey responses. Personalised reminder emails were sent to non-responders. This survey explored the frequency of patient input in COS studies, who was involved, what methods were used and whether or not the COS development was international.

**Results:**

One hundred and ninety-two COS developers were sent the survey. Responses were collected from 21 February 2017 until 7 May 2017. One hundred and forty-six unique developers responded, yielding a 76% response rate and data in relation to 195 unique COSs (as some developers had worked on multiple COSs). Of focus here are their responses regarding 162 COSs at the published, completed or ongoing stages of development. Inclusion of patient participants was indicated in 87% (141/162) of COSs in the published completed or ongoing stages and over 94% (65/69) of ongoing COS projects. Nearly half (65/135) of COSs included patient participants from two or more countries and 22% (30/135) included patient participants from five or more countries. The Delphi survey was reported as being used singularly or in combination with other methods in 85% (119/140) of projects. Almost a quarter (16/65) of ongoing studies reported using a combination of qualitative interviews, Delphi survey and consensus meeting.

**Conclusions:**

These findings indicated that the Delphi survey is the most popular method of facilitating patient participation, while the combination of qualitative interviews, Delphi survey and consensus meetings is the most popular combination of methods. The increased inclusion of patient participants in the development of COSs is encouraging, as is the international approach to COS development that some developers are adopting.

**Electronic supplementary material:**

The online version of this article (10.1186/s13063-018-2493-y) contains supplementary material, which is available to authorized users.

## Background

Evidence enables treatment decisions to be made according to the needs of the individual patient. This evidence comes from numerous studies that record and measure the effects that different illnesses, conditions and treatments have on patients. These measurements are known as ‘outcomes’. Outcomes can include quality of life, treatment costs, fatigue, white blood cell count and pain. However, across different studies of the same condition or illness there is considerable variability in the outcomes measured. This has given rise to difficulties such as: summarising the evidence as the results cannot be adequately compared and contrasted [1] and authors selectively reporting outcomes [2]. There is also the possibility that outcomes currently being measured may not accurately reflect the priorities of relevant stakeholder groups, including patients and the public. In turn, the usefulness of studies in advancing research, informing clinical practice and empowering clinicians and patients with knowledge regarding interventions is limited [3], rendering the research wasteful in many instances [4, 5].

One answer to this problem is the development of core outcome sets (COSs). A COS is a minimum set of agreed standardised outcomes which should be measured and reported in all trials of a specific condition. It is considered a fundamental list of outcomes [6], not an exhaustive list, and researchers can measure additional outcomes in their trials if they wish [3]. The same set may also be relevant to systematic reviews of trials. The Core Outcome Measures in Effectiveness Trials (COMET) Initiative was launched in 2010 in response to the recognised value of COS development. COMET aims to tackle the problem of heterogeneity in reported outcomes by promoting and facilitating the development and application of COSs. COMET also collates and stimulates the production of resources for COS development, and facilitates the exchange of ideas and methodological research to enhance the quality and uptake of COSs. COMET’s key resource is a publicly accessible database (www.comet-initiative.org) of planned, ongoing and completed COS studies. The database of published COSs is updated annually via a systematic review. As indicated by the multiple individuals and organisations, including trialists, funders, registries, regulatory authorities, systematic review groups and journal editors now endorsing the uptake of COSs and use of the COMET database (a list of these organisations is available at: www.comet-initiative.org/cosuptake), the usefulness and importance of this resource, and COS development more generally, is accepted.

Three stakeholder groups who are important to the development of all COSs are those who will use the COSs in research, health professionals and patients, as recently identified by the consensus-based recommendations in the Core Outcome Set – STAndards for Development: The COS-STAD recommendations [7, 8]. Thus, the inclusion of patients and the public is key to the development of COSs [9].

Moreover, to increase reporting completeness and transparency in COS development COMET have produced the Core Outcome Set – STAndards for Reporting COS-STAR checklist. This checklist states that authors should describe the participant groups involved, the rationale for including them and the capacity in which they participated in the COS development [10].

When using the term ‘patient’ in this article we refer to patients, carers, health and social care service users and people from organisations who represent these groups. Researchers are increasingly including patients alongside other stakeholders in identifying what outcomes to measure in clinical trials. While a 2013 systematic review found that only 18% of published COS studies reported patient input [11], subsequent updates of this review in 2014 and 2015 indicated patient input in 59% and 61% of published COS developments, respectively [12, 13]. For most conditions there are many different outcomes that could be included in a COS. When patients have not been included in the COS development process, important outcomes have been overlooked [14], while other evidence indicates that patients and families differ in the priority they give to certain outcomes compared to clinicians [15]. Including patient participants in deciding which of these outcomes should be in a COS reduces the danger of omitting important outcomes. More broadly, patient participation in COS development enhances the value of research as it helps to ensure that the outcomes reported are relevant to patients.

However, the best methods for facilitating the participation of patients is unknown. There are numerous challenges in enabling participation in a COS study and these will vary depending on the participants, the research team and the condition being researched. The COMET handbook brings together current thinking and methodological research regarding these challenges [7] including: selecting an appropriate recruitment method, finding the best way to explain the concept of a COS, using a suitable method to elicit perspectives of patients and health professionals, maintaining participant input over time, and enabling the inclusion of patients in face-to-face meetings with health professionals and academics [9]. Previous COS studies have reported variable rates of recruitment of participants in the development of the COS [12], while COS developers have also reported limited experience of engaging with participants in the development of important COSs [16].

This study examined the frequency of patient participation, which types of patient stakeholder are included, the methods employed to facilitate patient participation and the number of countries from which patients have been sampled in recently published and ongoing COS projects. By describing the current practice in the development of COSs, the survey will help to identify areas for further improvement and study.

## Methods

### Design

The survey was conducted in English and included some brief demographic questions before enquiring about patient participation in COS development. Patient participation was defined as: ‘where patients or the wider public (family members, carers, health and social care service users and people from organisations who represent these groups) or both, take part in the development of a core outcome set by giving data on their opinions regarding what outcomes are important.’ A full list of the questions can be found in Supplementary Information: Additional file 1. Survey Questions. The survey was constructed using the SurveyMonkey software [17]. The benefits of using this software include the facility to incorporate filtre questions (whereby depending on the responses, questions are automatically skipped to the next appropriate question), it also allowed flexibility in the answer options which was of vital importance to obtaining the responses in the most appropriate manner.

A survey was deemed appropriate for this particular phase of the project as it is inexpensive and allowed us to engage with a large number of COS developers. Studies have shown that questionnaire length has a substantial effect on the number of non-responders [18], this questionnaire was purposely kept short to avoid this issue and to not overburden any prospective respondents. Other factors thought to influence the overall response rate include readability of questionnaires, such as the number of syllables per word, words per sentence, typeface and font size. We therefore followed what is considered best practice in the academic literature (The National Institute of Adult Continuing Education guideline ‘Readability: How to produce a clear written materials for a range of readers’) when building this survey [18].

### Participant selection and recruitment

The COS developers were identified via a search (2 February 2017) of all studies published from 2013 and ongoing COS projects in the COMET Initiative database.

The survey was sent to COS developers as a link within a personalised email, inviting them to visit the SurveyMonkey website where the survey was hosted. Adopting a personalised approach and follow-up contact with those who do not respond to the initial email has been suggested to increase the odds of response by more than a quarter [18]; therefore, personalised emails were sent and three further emails were sent to non-responders.

### Analysis of survey responses

Responses relating to published COS projects were validated by reading the appropriate publications where these were available, and emailing COS developers for clarification where necessary. The data were analysed descriptively.

## Results

The survey was sent to 192 COS developers. Some developers were involved in multiple COS projects and we asked them to complete the survey for each relevant COS. We contacted 59 developers for 59 published COS projects, 129 developers for 150 ongoing COS projects and 4 developers for 16 published and 19 ongoing COS projects. Responses were collected from February until May 2017.

There were 146 respondents yielding a 76% response rate and providing data regarding 195 projects. The breakdown of respondents and their projects is as follows: 37 responders for 37 published COS projects, 29 responders for 29 completed COS projects, 49 responders for 52 ongoing COS projects, 25 responders for 27 planned COS projects, 6 responders for a mixture of 15 published, 12 completed, 17 ongoing and 6 planned COS projects.

Table 1 summarises the frequency of patient participation in 162 COS projects since 2013, from published, completed and ongoing studies, after excluding 33 studies still in the planning stage. Overall, respondents indicated that 141/162 (87%) COS projects had included patient participants in the development of their COS (Table 1).

**Table 1:** Frequency of patient participation in core outcome set (COS) projects by COS development stage

Stage of COS development	COS with no patient participants *n* (%)	COS including patient participants *n* (%)
Published	14 (27)	38 (73)
Completed	3 (7)	38 (93)
Ongoing	4 (6)	65 (94)
Total	21 (13)	141 (87)

Survey responses for patient participation matched published information for 51 COSs; in the remaining published study it was not possible to make this comparison as the developer did not provide their name in their survey response. Of 24 published COSs for which no survey response was received or could be matched, five (21%) of the journal articles reported patient participation.

Developers reported input from a variety of patient stakeholder groups (Table 2): 101 (72%) projects included both patients (healthcare patients, healthcare users, consumers, family members, spouse, carers, etc.) and patient organisations (patient support groups and patient charity representatives).

**Table 2:** Frequency of the patient participant groups included in core outcome set (COS) projects by COS development stage

Stage of COS development	COS including patient participants (*n* = 140)^a^		
	Patients and patient organisations *n* (%)	Patients only *n* (%)	Patient organisations only *n* (%)
Published	23 (62^a^)	14 (38)	0
Completed	28 (74)	10 (26)	0
Ongoing	50 (77)	14 (21)	1 (2)
Total	101 (72)	38 (27)	1 (1)

^a^No further information was provided in relation to one published study thus it has been excluded from further analysis

For projects including patient participants, Table 3 shows how many countries were involved in the 135 studies where a response was provided. Half of COS projects included patient participants from only one country, and this was usually the United Kingdom (41/70, 59%). Where the study was international, typically COS developers involved participants from five or more countries (*n* = 30/135, 23% of total COSs).

**Table 3:** How many patient participant countries are included in core outcome set (COS) development by COS development stage

Stage of COS development (*n*)	1 country *n* (%)	2 countries *n* (%)	3 countries *n* (%)	4 countries *n* (%)	5 + countries *n* (%)
Published (36)	21 (58)	5 (14)	1 (3)	1 (3)	8 (22)
Completed (36)	13 (36)	6 (17)	5 (14)	3 (8)	9 (25)
Ongoing (63)	36 (57)	10 (16)	2 (3)	2 (3)	13 (21)
Total (135)	70 (52)	21 (16)	8 (6)	6 (4)	30 (22)

Table 4 summarises COS developers’ responses regarding the methods that they had used to facilitate patient participation. Developers responded via a fixed response option that included five commonly used methods (Delphi survey, questionnaire, focus group, qualitative interview and consensus meeting) and an additional ‘other’ option, which prompted respondents to state the method in a free-text box. All method combinations can be found in Supplementary Information: Additional file 2. Method Combinations.

**Table 4:** shows the methods used either singularly or in combination to facilitate patient participation. A full breakdown of the methods can be found in Supplementary Information: Additional file 2. Method Combinations

Methods used	Published *n* (%)	Completed *n* (%)	Ongoing *n* (%)	Combined *n* (%)
Number of COS studies included	37	38	65	140
Delphi survey only	12 (32)	7 (18)	2 (3)	21 (15)
Questionnaire only	2 (5)	0	1 (2)	3 (2)
Qualitative interviews only	0	0	2 (3)	2 (1)
Consensus meeting only	2 (5)	0	0	2 (1)
Focus group only	0	1 (3)	0	1 (1)
Nominal group technique only	0	0	1 (2)	1 (1)
Mixed methods *(see descriptions below)*	21 (58)	30 (79)	59 (90)	110 (79)
*Delphi survey and another method(s)*	*15 (71)*	*26 (87)*	*56 (95)*	*97 (88)*
*Consensus meeting and another method(s)*	*6 (29)*	*2 (7)*	*2 (3)*	*10 (9)*
*Qualitative interview and another method(s)*	*0*	*1 (3)*	*1 (2)*	*2 (2)*
*Focus group and another method(s)*	*0*	*1 (3)*	*0*	*1 (1)*

*COS* core outcome set

As Table 4 shows, the Delphi survey was the most popular method, having been used singularly or in combination with other methods in over 119 (85%) of the 140 projects with patient participation. A multiple methods approach was used in 110 (79%) of the 140 projects with patient participation, of which the most popular method of was the combination of (1) Delphi survey, qualitative interviews and consensus meeting (22/140, 16%), followed by (2) Delphi survey singularly (21/140, 15%). In ongoing studies the most popular methods used were the combinations of (1) Delphi survey, consensus meeting and qualitative interviews (16/65, 25%), followed by (2) Delphi survey, consensus meeting, focus group and qualitative interviews (9/65, 14%) and finally (3) Delphi survey and consensus meeting (7/65, 11%).

## Discussion

This survey indicates that COS developers are increasingly including patients as participants in COS project development despite reports of COS developers finding patient participation difficult to facilitate in comparison to the participation of other stakeholder groups [16].

While many will welcome the increased inclusion of patients and patient organisations in COS development, it could also be argued that patient participants should exclusively be people who have personal experience of the condition or situation, as they are best placed to offer insight into what outcomes are important to someone living with a condition. This would exclude people working for patient organisations as COS study patient participants or others without personal experience of what it is like to live with a condition, as their perspectives may be closely aligned with that of a healthcare professional or researcher. Further research could examine what should constitute patient participation in COS development and explore the roles that these groups have and the similarities and differences in the input they provide.

The principle behind the development of a COS is that all researchers working on the same condition, illness or treatment will use that COS in their research. Therefore, COSs need to be relevant for use across different countries if they are to improve the power of research to benefit patients [9]. The findings of this survey are encouraging, with several COS projects being run in two or more countries with patient participants. However, the majority of COS projects mainly included patient participants from only one country, usually the UK. Previous research has indicated that COS developers have concerns regarding the practicalities and resources surrounding international COS development. Concerns were also raised in relation to ‘heterogeneity of views that might arise when participants are included from multiple countries’ [16]. Future research could explore methods of developing COSs with patients and health professionals from multiple countries in a practical and feasible manner.

A key challenge in patient participation is enabling patients to contribute their perspectives in ways that are meaningful and sustainable. It is vital that the methods suit the patient group concerned. Patient and public involvement where patients and the public are involved as active research partners in a COS project, can provide a patient and public perspective on the suitability of different methods from the design to conclusion of a COS project. The collaboration of researchers and patient and public involvement partners can help to ensure the appropriate design and conduct of a COS project. The survey responses indicated that the use of combinations of different methods, such as the Delphi survey, questionnaires, interviews, focus groups and consensus meetings, is not unusual. It was also evident that the Delphi survey was the most popular of all methods of participation in COS development. Delphi surveys can widen patient participation, promote transparency and offer anonymity. However, these surveys can be lengthy, and some believe these are intimidating for patient participants [9]. COS developers have acknowledged a need for guidance on conducting Delphi surveys and consensus meetings [16]. The COS-STAD recommendations identify minimum standards that should be met during the COS development [8].

A strength of this study was the relatively high response rate. However, non-response bias is a potential issue within this survey. The validation work shows non-respondents for published projects had a lower patient participation rate than that of those who responded. This is likely to also be true for non-respondents of ongoing studies, resulting in an over-estimate of patient participation reported in the survey. Full and accurate reporting of COS projects, including details of patient participation, should continue to improve if developers use the recently published COS-STAR reporting guideline [10].

## Conclusion

The ongoing inclusion of patient participants in the development of COSs is encouraging, as is the international approach that some developers are adopting, despite the academic literature suggesting that there are barriers to be overcome in developing international COS projects.

## Additional files

Additional file 1: Survey Questions. (DOCX 21 kb)

Additional file 2: Method Combinations. (DOCX 14 kb)

## Abbreviations

COMET: Core Outcome Measures in Effectiveness Trials
COS: Core outcome set
