# Correction to: Survey indicated that core outcome set development is increasingly including patients, being conducted internationally and using Delphi surveys

**DOI:** 10.1186/s13063-018-2595-6

**Published:** 2018-08-08

**Authors:** Alice M. Biggane, Lucy Brading, Philippe Ravaud, Bridget Young, Paula R. Williamson

**Affiliations:** 1 Department of Biostatistics, University of Liverpool, Liverpool, UK; 2 INSERM, U1153 Epidemiology and Biostatistics Sorbonne Paris Cité Research Center (CRESS), Methods of Therapeutic Evaluation of Chronic Diseases (METHODS) Team, Paris Descartes University, Sorbonne Paris Cité, Paris, France; 3 Institute of Psychology Health and Society/North West Hub for Trials Methodology Research, University of Liverpool, Liverpool, UK; 4 Centre de Recherche Epidémiologie et Statistique, INSERM U1153, Paris, France; 5 Cochrane France, Paris, France; 6 Centre d’Épidémiologie Clinique, Hôpital Hôtel-Dieu, Assistance Publique-Hôpitaux de Paris, 75004 Paris, France; 7 Department of Epidemiology, Mailman School of Public Health, Columbia University, New York, NY USA; 8 Department of Psychological Sciences and MRC North West Hub for Trials Methodology Research, University of Liverpool, Liverpool, UK; 9 MRC North West Hub for Trials Methodology Research, Department of Biostatistics, University of Liverpool, Liverpool, UK

## Correction

In the original publication [1] the abbreviation for Core outcome sets (COS) was pluralized unnecessarily throughout the next. The original article was updated to rectify this error.
