# Supplementary material for: Survey indicated that core outcome set development is increasingly including patients, being conducted internationally and using Delphi surveys
Source: Trials. 2018 Feb 17;19:113. doi: 10.1186/s13063-018-2493-y (PMC5816387; doi:10.1186/s13063-018-2493-y)
Supplement: Supplementary file 1 — Survey Questions. (DOCX 21 kb) [file 13063_2018_2493_MOESM1_ESM.docx]

**Additional File 1. Survey Questions.**

**Exploring patient input in core outcome set (COS) development.**

Please answer the following questions in relation to the most recent COS study in which you have been involved.

**1) Name:**

**2) What is the current status of your core outcome set (COS) study?**

Published

Completed- COS is being currently written up, under review or in press

Ongoing- data collection has started and is currently in process or under analysis

Planning stages - data collection has not yet started

**Involvement:**is where patients and the public are involved as research partners, co-investigators, advisors, or team members of a COS study. In this role, typically they will help in the design and conduct of your COS study (e.g. sitting on the study management or steering group, advising on the participant recruitment strategy or commenting on patient information leaflets and survey materials).

**3) Have you/ do you plan to involve the public or patients (as a research partner, co-investigator, advisor, or research team member) in your COS study?**

Yes

No

**Participation:** is where patients or the public take part in the development of a core outcome set by giving data on their opinions regarding what outcomes are important (e.g. by completing a Delphi survey or taking part in interviews). We refer to people in this role as ‘patient participants’.

**4) Have you included/do you plan to include patient participants (i.e. completing your Delphi survey or taking part in qualitative interviews, attending consensus meetings) in your core outcome set study?**

Yes

No

**Questions 5-11 as follows relate to patient participation only.**

**5) Please indicate which of the following groups you included/plan to include as patient participants in the development of this COS (Please select all that apply.)**

Patients

Public

Patient support group/ patient charity representative

Other (Please specify):

**6) From what countries are your patient participants from?**

United Kingdom (UK)

United States of America (USA)

Australia

****Belgium

Canada

China

France

Germany

Ireland

Italy

Japan

Netherlands

Singapore

Spain

Other (Please specify):

**7) Please indicate which methods you used or intend to use to facilitate patient participation in your COS study. (Please select all that apply.)**

Delphi survey (a structured technique to reach consensus)

Questionnaire

Focus group

Qualitative Interview

Consensus Meeting

Other:

**8) How did you decide on the above methods (as indicated in Q.3) for facilitating patient participation in your COS study? (Please select all that apply.)**

Based on the literature

Own previous experience with same methods for COS development

Problems with other methods

Suited our situation and circumstances

Based on the resources available

Based on expert advice

Other (please specify):

**9) From where did you/do you intend to recruit the patient participants for your study? (Please select all that apply.)**

Health institutions/ centres e.g. National Health Service (NHS). Health Service Executive (HSE) etc.

Patient support/advocacy groups/ social media

Patient organisations/ charities

Word of mouth

Patient research partner (access to patients)

Other (please specify):

**10) How did you decide on the recruitment methods to use above in Q.5? (Please select all that apply.)**

Based on the literature

Own previous experience with same methods for COS development

Problems with other methods

Suited our situation and circumstances

Based on the resources available

Based on expert advice

Other (please specify):
