# Supplementary material for: Survey indicated that core outcome set development is increasingly including patients, being conducted internationally and using Delphi surveys
Source: Trials. 2018 Feb 17;19:113. doi: 10.1186/s13063-018-2493-y (PMC5816387; doi:10.1186/s13063-018-2493-y)
Supplement: Supplementary file 2 — Method Combinations. (DOCX 14 kb) [file 13063_2018_2493_MOESM2_ESM.docx]

**Additional File 2. Method Combinations**

| **Methods used** | **Total**  **n (%)** | **Published**  **n (%)** | **Completed**  **n (%)** | **Ongoing**  **n (%)** |
| --- | --- | --- | --- | --- |
| Number of COS studies included | **140** | 37 | 38 | 65 |
| Qualitative interviews, Delphi survey and consensus meeting | **22** (16) | 2 (5) | 4 (10) | 16 (24) |
| Delphi survey | **21** (15) | 12 (33)   \|  \| \| --- \| \|  \| | 7 (18) | 2 (3) |
| Focus group, questionnaire, Delphi survey and consensus meeting | **16** (11) | 2 (5) | 8 (20) | 6 (8) |
| Delphi survey and consensus meeting | **15** (11) | 1 (3) | 7 (18) | 7 (11) |
| Focus group**,** qualitative interviews, Delphi survey and consensus meeting | **10** (7) | 0 | 1 (3) | 9 (14) |
| Focus group, Delphi survey and consensus meeting | **7** (5) | 1 (3) | 0 | 6 (8) |
| Questionnaire, Delphi survey and consensus meeting | **6** (4) | 2 (5) | 1 (3) | 3 (5) |
| Questionnaire, qualitative interviews, focus group, Delphi survey and consensus meeting | **6** (4) | 1 (3) | 0 | 5 (6) |
| Qualitative interviews and Delphi survey | **5** (4) | 2 (5) | 2 (5) | 1 (2) |
| Delphi survey and focus group | **3** (2) | 0 | 2 (5) | 1 (2) |
| Questionnaire | **3** (2) | 2 (5) | 0 | 1 (2) |
| Qualitative interviews | **2** (1) | 0 | 0 | 2 (3) |
| Consensus meeting | **2** (1) | 2 (5) | 0 | 0 |
| Focus group, qualitative interviews and Delphi survey | **2** (1) | 0 | 1 (3) | 1 (2) |
| Qualitative interviews and consensus meeting | **2** (1) | 1 (3) | 0 | 1 (2) |
| Focus group, qualitative interviews and consensus meeting | **2** (1) | 2 (5) | 0 | 0 |
| Focus group and consensus meeting | **2** (1) | 1 (3) | 0 | 1 (2) |
| Questionnaire, qualitative interviews, Delphi survey and consensus meeting | **2** (1) | 2 (5) | 0 | 0 |
| Questionnaire, focus group and qualitative Interviews | **1** (1) | 0 | 0 | 1 (2) |
| Questionnaire, focus group**,** qualitative interviews and consensus meeting | **1** (1) | 0 | 1 (3) | 0 |
| Questionnaire and Delphi survey | **1** (1) | 1 (3) | 0 | 0 |
| Questionnaire, focus group and Delphi survey | **1** (1) | 1 (3) | 0 | 0 |
| Focus group | **1**  (1) | 0 | 1 (3) | 0 |
| Questionnaire, qualitative interviews and Delphi survey | **1**  (1) | 0 | 0 | 1 (2) |
| Questionnaire and qualitative interviews | **1**  (1) | 0 | 1 (3) | 0 |
| Focus group and questionnaire | **1**  (1) | 0 | 1 (3) | 0 |
| Focus group**,** qualitative interviews, Consensus meeting and other (nominal group technique) | **1**  (1) | 0 | 1 (3) | 0 |
| Other (nominal group technique) | **1**  (1) | 0 | 0 | 1 (2) |
| Other (nominal group technique) and consensus meeting | **1** (1) | 1 (3) | 0 | 0 |
| Focus group and other (group concept mapping) and consensus meeting | **1**  (1) | 1 (3) | 0 | 0 |

Table 4- Full Version shows the methods used to facilitate patient participation. The methods were used either singularly or in combination.
